# Functional Insights From the Evolutionary Diversification of Big Defensins

**DOI:** 10.3389/fimmu.2020.00758

**Published:** 2020-04-30

**Authors:** Marco Gerdol, Paulina Schmitt, Paola Venier, Gustavo Rocha, Rafael Diego Rosa, Delphine Destoumieux-Garzón

**Affiliations:** ^1^Department of Life Sciences, University of Trieste, Trieste, Italy; ^2^Laboratorio de Genética e Inmunología Molecular, Instituto de Biología, Pontificia Universidad Católica de Valparaíso, Valparaíso, Chile; ^3^Department of Biology, University of Padova, Padova, Italy; ^4^Laboratory of Immunology Applied to Aquaculture, Department of Cell Biology, Embryology and Genetics, Federal University of Santa Catarina, Florianópolis, Brazil; ^5^IHPE, Université de Montpellier, CNRS, Ifremer, Université de Perpignan Via Domitia, Montpellier, France

**Keywords:** antimicrobial peptide, defensin, evolution, nanonet, host defense (antimicrobial) peptides

## Abstract

Big defensins are antimicrobial polypeptides believed to be the ancestors of β-defensins, the most evolutionary conserved family of host defense peptides (HDPs) in vertebrates. Nevertheless, big defensins underwent several independent gene loss events during animal evolution, being only retained in a limited number of phylogenetically distant invertebrates. Here, we explore the evolutionary history of this fascinating HDP family and investigate its patchy distribution in extant metazoans. We highlight the presence of big defensins in various classes of lophotrochozoans, as well as in a few arthropods and basal chordates (amphioxus), mostly adapted to life in marine environments. Bivalve mollusks often display an expanded repertoire of big defensin sequences, which appear to be the product of independent lineage-specific gene tandem duplications, followed by a rapid molecular diversification of newly acquired gene copies. This ongoing evolutionary process could underpin the simultaneous presence of canonical big defensins and non-canonical (β-defensin-like) sequences in some species. The big defensin genes of mussels and oysters, two species target of in-depth studies, are subjected to gene presence/absence variation (PAV), i.e., they can be present or absent in the genomes of different individuals. Moreover, big defensins follow different patterns of gene expression within a given species and respond differently to microbial challenges, suggesting functional divergence. Consistently, current structural data show that big defensin sequence diversity affects the 3D structure and biophysical properties of these polypeptides. We discuss here the role of the N-terminal hydrophobic domain, lost during evolution toward β-defensins, in the big defensin stability to high salt concentrations and its mechanism of action. Finally, we discuss the potential of big defensins as markers for animal health and for the nature-based design of novel therapeutics active at high salt concentrations.

## Introduction

Host defense peptides (HDPs) comprise bioactive molecules produced by virtually all life forms. Initially characterized for their antimicrobial properties and accordingly named antimicrobial peptides (AMPs), they were described as peptides, usually cationic, which selectively target essential microbial components (1). More than natural antibiotics, HDPs perform a wide range of both immune and non-immune functions (2). Although every species has typically its own repertoire of HDPs, molecular evolution has led to the convergence on a few highly successful structural scaffolds widely distributed in multicellular organisms. Defensins probably represent the most striking example of this process, as they are found in nearly all multicellular Eukaryotes, from fungi and spermatophyte plants to animals (both Protostomia and Deuterostomia) (3).

**FIGURE 1:**
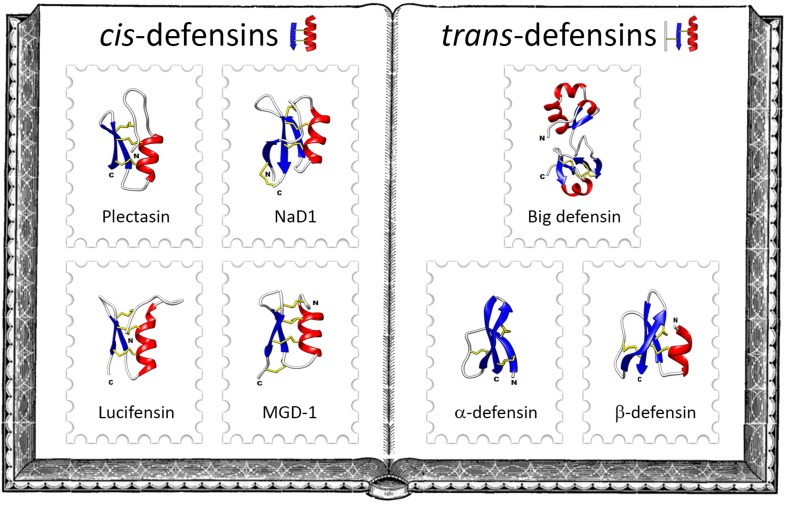
The family album of defensins. Left-hand side of the album illustrates some classical *cis*-defensins: the fungal defensin plectasin from the ebony cup mushroom *Pseudoplectania nigrella* (PDB: 1ZFU), the plant defensin NaD1from the flowering tobacco *Nicotiana alata* (PDB: 1MR4), the insect defensin lucifensin from the green bottle fly *Lucilia sericata* (PDB: 2LLD) and the mollusk defensin MGD-1 from the Mediterranean mussel *Mytilus galloprovincialis* (PDB: 1FJN). Right-hand side of the album exemplifies some members of the main families of *trans*-defensins: the big defensin *Cg*-BigDef1 from the Pacific oyster *Crassostrea gigas* (PDB: 6QBL), the α-defensin HD5 (PDB: 2LXZ) and the β-defensin hBD-1 (PDB: 1IJV) from humans. Protein Data Bank (PDB) numbers are indicated in parentheses, α-helices in red, β-strands in blue and disulfide bonds in yellow.

Defensins are gene-encoded disulfide-rich antimicrobial peptides (4). They are produced by various tissues according to species, and can be constitutively expressed or induced in response to different stimuli (infection, injury, inflammatory factors, etc.). Recent phylogenetic studies have classified defensins into two analogous superfamilies, namely *cis*-defensins and *trans*-defensins, that arose from different origins, but that underwent convergent evolution in terms of structure and function (5) (Figure 1). This classification is based on the spacing and pairing of the cysteine residues and the orientation of the peptide secondary structure. *Cis*-defensins contain two parallel disulfide bonds that stabilize the final β-strand to an α-helix (6). This folding is a key element in a 3D structure known as cysteine-stabilized α-helix/β-sheet (CSαβ) motif, which is shared by all *cis*-defensins as well as by plant trypsin inhibitors and scorpion neurotoxins (7). In *trans*-defensins, two disulfide bonds point in opposite directions from the final β-strand and stabilize different secondary structure elements (6). All *trans*-defensins share a conformational structure consisting of three anti-parallel β-strands stabilized by three disulfide bonds (8) but they adopt a diversity of 3D structures that do not systematically include α-helices (Figure 1). CSαβ-containing peptides from the *cis*-defensin superfamily may have six, eight or ten cysteines whereas *trans*-defensins contain six cysteine residues.

*Cis*-defensins are widely distributed across the fungal, plant and animal kingdoms. In contrast, *trans*-defensins have arisen and evolved exclusively in animals. Based on the disulfide bond arrangement of their six conserved cysteine residues and 3D structures, *trans*-defensins are subdivided into different families: α-defensins, β-defensins and big defensins (Figure 1). Aside from these families, a defensin with a cyclic peptide backbone was named θ-defensin; it is related to α-defensins and exists only in some non-human primates (9). α-defensins are peptides stabilized by the cysteine pairing Cys_1__–__6_Cys_2__–__4_Cys_3__–__5_ (4), they were the first group of defensins to be described. Originally isolated from rabbit granulocytes in 1984 (10), they have only been identified in a few mammalians. β-defensins are peptides holding three intramolecular disulfide bonds paired as Cys_1–5_Cys_2__–__4_Cys_3__–__6_. They occur from teleost fish to mammals and are considered as the oldest type of vertebrate defensin (11).

**FIGURE 2:**
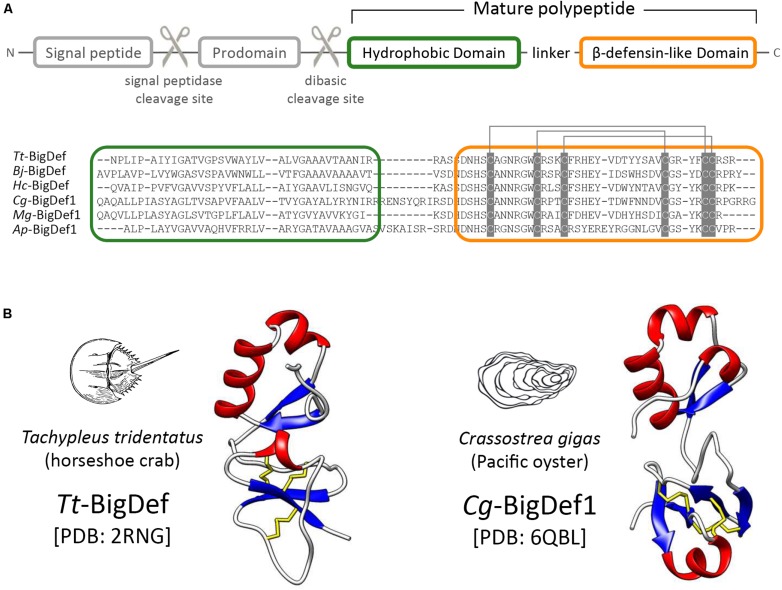
Structural domain organization of big defensins. **(A)** Big defensin precursors are composed of a signal peptide followed by a prodomain holding a dibasic cleavage site, and a multi-domain polypeptide (mature big defensin). Hydrophobic (green frame) and β-defensin like (orange frame) domains are indicated at the amino acid sequence alignment of certain mature big defensins. Cysteine pairing is indicated by gray lines. GenBank numbers are provided in Supplementary Table S1. **(B)** The 3D structure of canonical big defensins shows the two structural domains connected by a flexible linker.

The last family of *trans*-defensins known as big defensins was isolated from the hemocytes, i.e., circulating immune cells, of the horseshoe crab *Tachypleus tridentatus*, an ancient marine chelicerate (Merostomata) (12). Big defensins are composed of a C-terminal β-defensin-like domain combined with a hydrophobic globular N-terminal domain (Figure 2). The *T. tridentatus* big defensin (*Tt*-BigDef) is stored in hemocyte granules (13) and displays antimicrobial activities and LPS-binding properties (12). Homologs of *Tt*-BigDef have been identified in bivalve mollusks (Bivalvia) and amphioxus (Cephalochordata) by molecular approaches (14–17).

With few exceptions, big defensin precursors are synthesized as prepropeptides in which a prodomain is located downstream of the signal sequence (15) (Figure 2A). This prodomain, whose function remains unknown, ends with a conserved dibasic site (either Lys-Arg or Arg-Arg), which is likely recognized by a furin-like peptidase during big defensin maturation, like other invertebrate AMPs (18, 19) (Figure 2A). Additional post-translational modifications (e.g., oxidation of disulfide bonds, C-termination amidation) give rise to mature big defensins (15).

Mature big defensins harbor a N-terminal hydrophobic region and a C-terminal region that contains six cysteines (Figure 2A). To date the horseshoe crab *Tt-*BigDef and the Pacific oyster *Cg-*BigDef1 are the only two big defensins for which a three-dimensional structure has been obtained (20, 21) (Figure 2B). Both molecules are highly soluble in solution. They are composed of two distinct globular domains connected by a flexible linker. Their hydrophobic N-terminal domain adopts a β1-α1-α2-β2 fold while their cationic C-terminal domain shows the cysteine pairing of β-defensins (Cys_1–5_Cys_2__–__4_Cys_3__–__6_). The flexible linker is longer in *Cg-*BigDef1 than in *Tt-*BigDef, which determines a different orientation of the N- and C-terminal domains in each molecule (Figure 2B). Basic or dibasic sites (Arg-Arg or Lys-Arg) are found between the two structural domains of big defensins. The proteolytic cleavage of the native *Tt*-BigDef at this dibasic site, experimentally achieved (12), generated two fragments with distinct antimicrobial activities, as also observed for the two synthetic domains of *Cg*-BigDef1 (21). The covalent association of *Cg-*BigDef1 domains is synergistic and essential for salt-stable antimicrobial activity (21).

The discovery of big defensins has rekindled the discussion about the evolutionary history of *trans*-defensins (22). Both structural and phylogenetic studies have provided compelling evidence that big defensins could be the missing link in vertebrate defensin evolution, as an invertebrate big defensin gene has been hypothesized as the most probable ancestor of present-day β-defensins (5, 22). It is noteworthy that the N-terminal hydrophobic region is the hallmark of big defensins, a trait that was lost during the transition from basal chordates to their vertebrate relatives (22). In the subsequent sections we explore the taxonomic distribution and extraordinary diversification of the big defensin family in terms of sequence, tissue expression, gene regulation and mechanism of action. We discuss the functional meaning of the N-terminal domain conservation and translational insights that can be gained from a functional perspective.

## Preamble

In this review, we discuss the molecular diversity and biochemical properties of big defensin sequences subject of previous studies and deposited in publicly available repositories. However, to provide a more comprehensive overview of the taxonomic distribution of these HDPs, we extend our investigation to several large, but still unexplored phyla, for which genomic or taxonomic resources are available. Consequently, while all the big defensin sequences described in this review derive from the screening of previously published sequence data, most of them had not been formally identified or described before. The big defensin sequences described in this article, with IDs and references, are reported in Supplementary Table S1.

Our approach was based on *in silico* data mining and exploited the conserved phylogenetic signal shared by all big defensins. In brief, known big defensin sequences were retrieved from the NCBI nr protein database and the redundancy of the dataset was reduced with CD-HIT v4.6.8 (23), based on a pairwise sequence identity threshold of 60%. The multiple sequences alignment obtained with MUSCLE (24) was used to generate a Hidden Markov Model profile for HMMER v3.3 (25). This profile was used to screen the genomes and transcriptomes of the species mentioned in the following sections based on an *e*-value threshold of 1E^−3^. In detail, gene annotations, whenever available, were used to obtain protein predictions from genomes, and TransDecoder v5.5.0 was used to virtually translate transcriptomes. tBLASTn^1^ was used as a complementary tool for the identification of unannotated genes, using an *e*-value threshold of 1E^−3^. All retrieved hits were manually curated and the approach was re-iterated, by regenerating the HMM profile, until no new hits could be found.

The results here presented are largely dependent on the availability of -omic resources for the screening, on the completeness of the transcriptomes that we analyzed and on the quality of the genome assemblies and annotations. Therefore, our inference about the presence or absence of big defensins in a given taxa, as well as the estimates of the number of paralogous genes per species are subject to future update and revision.

## The Broad but Discontinuous Taxonomic Distribution of Big Defensins

### Ecdysozoa (A Large Monophyletic Group of Invertebrate Animals Belonging to Protostomia, Which Undergo Molting, e.g., Arthropods, Nematodes, and Other Minor Phyla)

**FIGURE 3:**
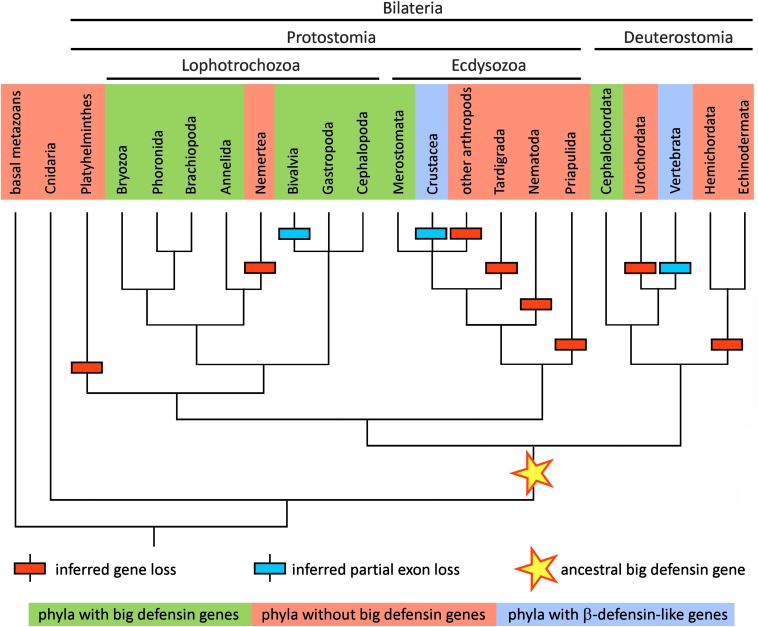
Schematic representation of the metazoan tree of life, reporting the presence of big defensins in all the major phyla relevant in the context of this study. The topology of the tree follows the branching pattern resulting from a recent phylogenomic study (100). Taxa where big defensins have been reported are marked with a green background and those where big defensins are absent are marked with a red background. Taxa where no big defensins are present, but β-defensin-like peptides have been reported, are indicated with a light blue background. The hypothetical origin of the primordial big defensin gene is shown with a star. Inferred big defensin gene loss events are indicated with a red box and inferred partial exon loss events are indicated with a blue box.

Although 25 years have passed since the initial discovery of big defensins in *T. tridentatus* (12, 13), horseshoe crabs (class Merostomata) still remain the only clade of arthropods where these HDPs have been formally described. Indeed, while orthologous sequences are expressed in the transcriptomes of the two other extant genera of horseshoe crabs, i.e., *Limulus* and *Carcinoscorpius*, no trace of big defensins has ever been found in insects, arachnids and crustaceans, in spite of the high amount of -omic data available. Based on the analysis of fully sequenced genomes, this consideration can be further extended to the Tardigrada, Nematoda and Priapulida, which points out a very narrow taxonomic distribution of big defensins within Ecdysozoa (Figure 3), the largest group of animals, with over 4.5 million estimated extant species (26).

The only other known case of peptides bearing a β-defensin-like cysteine array in Ecdysozoa is that of panusins, a family of HDPs which have been first identified in a crustacean, the spiny lobster *Panulirus* spp. (27, 28). In spite of a significant primary sequence homology with the C-terminal domain of big defensins, panusins are completely devoid of the N-terminal region and more closely resemble the architecture of vertebrate β-defensins. A similar sequence was recently identified in another decapod crustacean, the lobster *Homarus americanus* (29).

### Lophotrochozoa (A Large Monophyletic Group of Invertebrate Animals Belonging to Protostomia That Share the Lophophore Feeding Structure and the Trochophore Developmental Stage, e.g., Mollusks, Annelids, and Many Other Minor Phyla)

In stark contrast with the scarce number of reports in Ecdysozoa, big defensins have been found on multiple occasions in Lophotrochozoa. They have been described in nearly all lineages of Bivalvia (Mollusca), mostly including marine species of mussels (16), scallops (14, 30, 31), oysters (15), clams (32, 33) and ark shells (34), but also in a freshwater species belonging to the family Unionidae (35). While no big defensin has been formally reported in the other molluscan classes, the results of our screening suggest that the phylogenetic spread of these HDPs in Mollusca extends far beyond bivalves (Figure 3).

In spite of the relevant amount of -omic resources available for Gastropoda (which include over 80, 000 classified species of snails and slugs) (36), we could identify big defensins only in abalones and in a few snails, which suggests that these HDPs are likely to be present only in some (but not all) species. The existence of big defensins in cephalopods (e.g., octopuses and squids) is supported by both genomic and transcriptomic evidence: while the only sequence deposited in public databases is a mRNA expressed in the photophore of the squid *Pterygioteuthis hoylei*, we could identify unannotated big defensin orthologs in the genomes of *Octopus* spp. and *Architeuthis dux*. Moreover, a big defensin transcript was also detected in *Chiton olivaceus*, a species belonging to a minor molluscan class (Polyplacophora).

Very fragmentary information is available for the other lophotrochozoan phyla, most likely due to the limited -omic resources available and to the lack of efforts specifically focused on the study of AMPs in these organisms. Big defensins have been previously evidenced in Rhynchonelliformea, one of the three subphyla of the phylum Brachiopoda (37). Here we can also report the presence of big defensins in the transcriptomes of several other distantly related lophotrochozoan species, which include the bryozoan *Flustra foliacea*, two species of sabellid polychaetes (Annelida), and two congeneric species of phoronids. On the other hand, the genomes of many other lophotrochozoans, such as the annelids *Capitella teleta* and *Helobdella robusta*, or the ribbon worm *Notospermius geniculatus*, as well as the genomes of the early branching spiralian groups (e.g., Platyhelminthes, Rotifera and Gastrotricha) are completely devoid of big defensin genes, confirming the scattered distribution of these HDPs in metazoans (Figure 3).

### Deuterostomia (i.e., the Sister Group of Protostomia, Characterized by a Different Embryonic Development. This Group Includes, Among the Others, Echinoderms, Tunicates, Amphioxi, and Vertebrates)

Among deuterostomes, big defensins have been only found in Cephalochordata (amphioxi, or lancelets). The cloning of a big defensin cDNA from *Branchiostoma japonicum* (17) finds full support in the presence of orthologous sequences in the genomes of the other cephalochordate species *Branchiostoma belcheri*, *Branchiostoma floridae* and *Asymmetron lucayanum*. On the other hand, big defensins are apparently present neither in Ambulacraria (Hemichordata + Echinodermata) nor in Urochordata (Figure 3). Even though vertebrates do not display big defensins, they possess a related family of *trans*-defensins named β-defensins (38). These defense peptides underwent a remarkable diversification in vertebrates in which they spread from teleosts to mammals (39). Although they share an identical pairing of cysteines, they entirely lack the N-terminal region typical of big defensins.

## Big Defensins, β-Defensins and Panusins: a Shared Evolutionary Origin?

The phylogenetic distribution of big defensins suggests that these sequences are monophyletic and derive from a primordial big defensin gene already present in the latest common ancestor of all bilaterian animals, before the speciation process that gave rise to protostomes and deuterostomes (Figure 3). However, the timing of the appearance of the first big defensin gene is presently unclear, since no big defensin or any other *trans*-defensin-encoding genes have ever been described in extant representatives of early branching metazoan phyla (e.g., Porifera, Cnidaria, Ctenophora, etc.). Nevertheless, the scattered distribution of these molecules in the animal phylogeny may seem counterintuitive and requires some explanation. Over long evolutionary timescales, gene death occurs with high frequency (40), contributing to animal genetic and phenotypic variation (41). The multiple independent rounds of lineage-specific gene contraction/loss events documented along metazoan evolution (42, 43) may be fully consistent with the discontinuous taxonomic distribution of big defensins (Figure 3).

**FIGURE 4:**
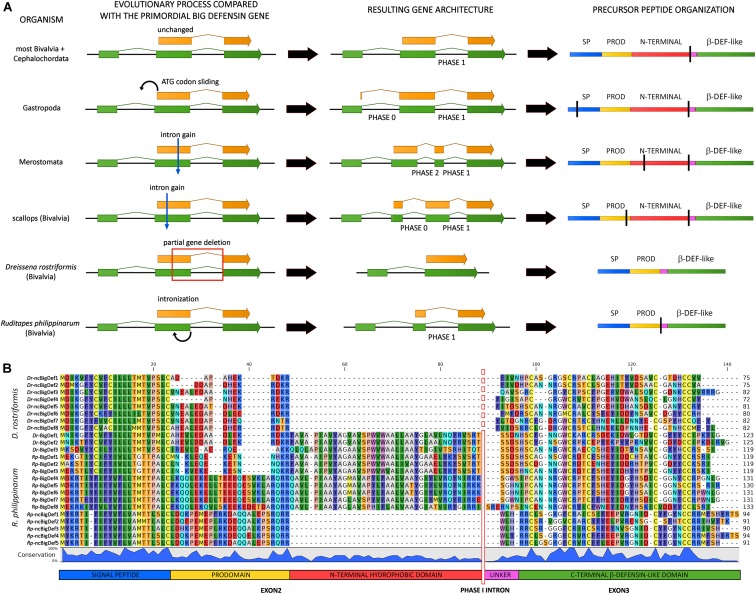
Inferred evolutionary processes that may have led to the big defensin gene architecture observed in extant metazoan taxa. **(A)** mRNAs are indicated by green arrows and protein-coding regions are indicated with orange arrows. On the right-hand side, a schematic organization of the encoded precursor peptides is shown, including the signal peptide (SP), prodomain (PROD), N-terminal hydrophobic domain, linker and C-terminal β-defensin-like domain. Vertical black bars highlight the positioning of introns. **(B)** Multiple sequence alignment of the canonical (BigDefs) and non-canonical (ncBigDefs) big defensin peptides identified in the genomes and transcriptomes of *Dreissena rostriformis* and *Ruditapes philippinarum*. The organization of the main regions of the precursor peptides is shown at the bottom of the figure. The location of the phase I intron is indicated by a vertical bar (dashed in *Dr*-ncBigDefs, where it was lost). Note the large deletion of the N-terminal region which characterizes the non-canonical genes of both species.

A key question that remains to be answered is whether big defensins are evolutionarily related with vertebrate β-defensins and crustacean panusins, or the similarity in the disulfide array of these peptides is rather the product of convergent evolution. Zhou and Gao provided compelling evidence in support of a shared evolutionary origin for vertebrate β-defensins and invertebrate big defensins (22). Both gene types share a phase I intron (i.e., with the splicing site placed between the first and the second position of a codon) in a highly conserved position, at the 5′ end of the region encoding the C-terminal cysteine-rich region. The conservation of gene structure and intron phase are both considered important indicators of shared ancestry among distantly related genes (44). This is further supported by the recent release of horseshoe crab (*Limulus polyphemus*) and gastropod (*Pomacea canaliculata*) genomes in which big defensin genes share the very same highly conserved phase I intron (Figure 4A).

Most big defensin genes are characterized by the presence of three exons, with the coding region being split between the second and the third exon, as in the case of most bivalves and amphioxus (Figure 4A). However, several exceptions to this general and likely ancestral gene architecture exist. For example, the position of the initial ATG codon slid back into exon 1 in gastropods, leading to the creation of a phase 0 intron (Figure 4A). Moreover, the big defensin genes of horseshoe crabs and scallops display an additional intron (found in phase 0 and phase 2, respectively), which splits exon 2 in two smaller exons (Figure 4A).

Different genetic mechanisms may explain the divergent structure of the precursor peptides encoded by invertebrate big defensins and vertebrate β-defensins. Zhou and Gao (22) proposed two equally plausible alternative hypotheses to explain the loss of the N-terminal region in the vertebrate lineage: (i) partial intronization of exon 2; (ii) exon shuffling and combination of the 3′ exon, encoding the cysteine-rich module, with diverse upstream leader regions.

The genomes of two bivalve mollusks, the Manila clam *Ruditapes philippinarum* (45) and the zebra mussel *Dreissena rostriformis* (46), may represent cornerstones for understanding the molecular mechanisms behind the generation of genes encoding β-defensin-like peptides from a canonical big defensin gene. Indeed, both species display the simultaneous presence of canonical big defensins (carrying the N-terminal hydrophobic domain typical of this peptide family) and shorter non-canonical peptides (lacking this domain), which are characterized by large indels (∼40 amino acids) and resemble vertebrate β-defensins and crustacean panusins (Figure 4B) (33). As suggested by phylogenetic inference (see the following section), these two types of sequences are likely encoded by paralogous genes, ruling out the possibility of their origin by exon shuffling. The genetic mechanisms that led to the loss of the N-terminal region in *Ruditapes* and *Dreissena* are, however, largely different. In fact, the presence of a phase 1 intron and the contemporary presence of a short exon 2 in the Manila clam would be fully consistent with the intronization hypotheses proposed by Zhou and Gao (22) (Figure 4A). On the other hand, the non-canonical big defensin zebra mussel genes entirely lack intron 2 and therefore only display a single uninterrupted open reading frame, which is entirely embedded in the second exon (Figure 4A). This observation strongly suggests that the loss of the N-terminal region in *Dreissena* was not driven by intronization, but rather by the deletion of the genomic region comprising the 3′ end of exon 2 along with the entire intron 2, paired with the in-frame rejoining between the remnant part of exon 2 and exon 3. Unlike panusins in decapods and β-defensins in vertebrates, this evolutionary process acted on paralogous gene copies, maintaining the original canonical big defensin genes intact.

Altogether, these observations highlight that different genetic mechanisms may have independently originated β-defensin-like molecules using canonical big defensin genes as templates in vertebrates, crustaceans (i.e., panusins), bivalves and possibly other unexplored taxa.

## Inter- and Intra-Specific Sequence Diversity: Bivalves as a Case Study

**FIGURE 5:**
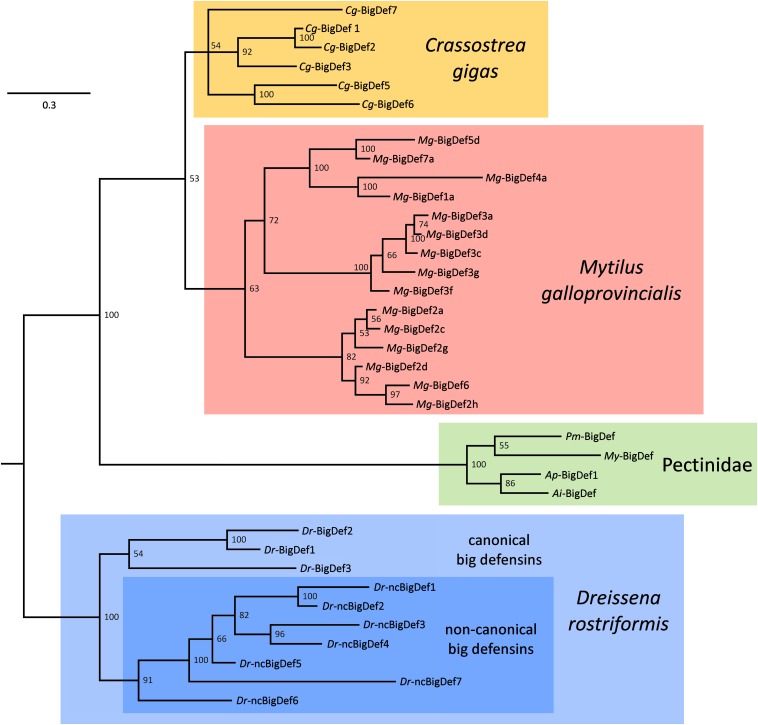
Simplified phylogeny of bivalve big defensins, exemplified by variants found in the genomes and transcriptomes of the oyster *Crassostrea gigas* (*Cg*-BigDefs), the mussel *Mytilus galloprovincialis* (*Mg*-BigDefs), the clam *Dreissena rostriformis* (*Dr*-BigDefs and (*Dr*-ncBigDefs) and the four scallop species *Pecten maximus* (*Pm*-BigDef), *Mizuhopecten yessoensis* (*My*-BigDef), *Argopecten purpuratus* (*Ap*-BigDef1) and *Argopecten irradians* (*Ai*-BigDef). The tree was obtained through Bayesian inference using MrBayes v3.2.1, with two parallel MCMC analyses run for 500, 000 generations each, based on a VT + G model of molecular evolution. For simplicity’s sake, variants sharing >90% pairwise identity have been removed. The tree was rooted on *D. rostriformis*, as the only member of the superorder Imparidentia. Posterior probability support values are shown for each node.

Due to the abundant literature on big defensins and the good number of fully sequenced genomes available, bivalves represent an excellent case study for investigating the processes behind the remarkable primary sequence diversity observed, both between and within species (Figure 5).

Quite surprisingly, bivalve genome data support the presence of a highly variable number of big defensin genes per species, ranging from zero to several copies. In line with the hypothesis of multiple independent rounds of lineage-specific gene loss, a few bivalve species are completely devoid of big defensins (e.g., *Modiolus philippinarum*, *Sinonovacula constricta*, and *Lutraria rhynchaena*), or only show relict pseudogenes with in-frame stop codons (e.g., the pearl oyster *Pinctada fucata*). Other bivalve species, such different scallops, the deep-sea hydrothermal vent mussel *Bathymodiolus platifrons* and the ark shell *Scapharca broughtonii* only carry a single functional big defensin gene. In contrast, many bivalve species retain two or more potentially functional big defensin genes, such as the freshwater mussel *Venustaconcha ellipsiformis*, with two paralogous gene copies, and *R. philippinarum*, with four (two canonical and two non-canonical big defensin genes, respectively).

A particularly complex situation can be observed in oysters whose genomes usually bear multiple big defensin genes. Nine out of the ten genes found in the reference genome of the Eastern oyster (*Crassostrea virginica*) are found in two distinct clusters of tandemly duplicated genes located on chromosome 2, containing 5 and 4 genes each. In a similar fashion, the genome of the Sidney rock oyster *Saccostrea glomerata* is characterized by the presence of six tandemly duplicated big defensin gene models, organized in a single cluster. In both oyster species, the precursor peptides encoded by these gene clusters display a highly variable level of pairwise sequence identity, which ranges from over 90% to as low as ∼25%, suggesting very different timings for the underlying gene duplication events. While at least three different big defensin genomic sequences have been described in the Pacific oyster *Crassostrea gigas* (15), the recent release of novel genomic data suggests that the big defensin gene repertoire of this species may be even larger (47).

Another example of a bivalve species bearing multiple big defensin genes is the zebra mussel *D. rostriformis*, which only shows a single gene encoding a canonical big defensin (a second copy is a pseudogene), and four tandemly duplicated non-canonical genes. Finally, the genome of the Mediterranean mussel *Mytilus galloprovincialis* contains six paralogous big defensin genes, which are mostly scattered in different genomic locations and encode proteins with different levels of pairwise similarity (ranging from ∼45 to over 90%) (48).

Although this has not been established yet, recurrent gene conversion among recently duplicated paralogs may explain, at least to some extent, the high level of intraspecific sequence variation of big defensins, mirroring the case of some insect AMPs, like attacins and diptericins (49, 50). The intricate evolutionary scenario of bivalve big defensin genes can be only in part disentangled with the aid of phylogenetic inference. Here we present a highly simplified overview of the relationships between the sequences identified in the mussel *M. galloprovincialis*, in the oyster *C. gigas*, in the freshwater mussel *D. rostriformis* and in four scallop species (Figure 5). Although some uncertainties remain due to the presence of some poorly supported nodes, the topology of the tree enables to assert that:

(i) All the different variants found in the same species appear to be monophyletic, suggesting an origin by independent species-specific gene family expansion events, driven by tandem gene duplication and, possibly, gene conversion among paralogs;
(ii) Gene duplication has often been followed by a fast process of molecular diversification, as evidenced by the high diversity of the variants found in *C. gigas*, *M. galloprovincialis* and *D. rostriformis*;
(iii) The magnitude of intraspecific big defensin sequence diversity often exceeds interspecific diversity, as highlighted by the comparison between the three aforementioned species and the four scallop orthologs;
(iv) The *D. rostriformis* canonical and non-canonical big defensin genes are monophyletic, which reinforces the hypothesis concerning the shared evolutionary origins of these HDPs;
(v) Altogether, these observations suggest that all bivalve big defensins have originated from a single ancestral gene, which was maintained in a single copy with little variation is some taxa (e.g., Pectinidae) or underwent repeated duplications and fast diversification in others.

The gene presence/absence variability (PAV) phenomenon (which indicates the presence of a gene in some, but not all the individuals belonging to the same species), adds a further layer of complexity to the highly dynamic genomic context outlined above. A growing body of evidence indicates that PAV is pervasive in some bivalve species, such as mussels, where it often targets HDP-encoding gene families (48). PAV most certainly shapes the individual repertoire of big defensins in *C. gigas*, as revealed by the patterns of presence/absence documented by PCR in 163 specimens (51) (Supplementary Figure S1). Although this situation would be potentially compatible with the presence of a single big defensin gene characterized by three highly polymorphic alleles (*Cg*-BigDef1, *Cg*-BigDef2, and *Cg*-BigDef3), the release of two complete genome assemblies (47, 52) revealed that the Pacific oyster, like the congeneric species *C. virginica*, most certainly holds multiple big defensin gene copies.

The data recently collected from the analysis of the *M. galloprovincialis* genome provide further data in support of the relevance of PAV in the context of big defensin intraspecific sequence diversity. Overall, a total of 33 unique variants, belonging to six sequence clusters (*Mg*-BigDef1, *Mg*-BigDef2/6, *Mg*-BigDef3, *Mg*-BigDef4, *Mg*-BigDef5, and *Mg*-BigDef7), were identified in 15 resequenced individuals. Although this categorization did not always allow to discriminate between paralogous genes and allelic variants encoded by the same genomic locus (e.g., up to four variants per cluster were found in some mussels), it allowed to ascertain that mussel big defensin genes are frequently subject to PAV (Supplementary Figure S1).

## Polymorphism of Big Defensin Expression

**FIGURE 6:**
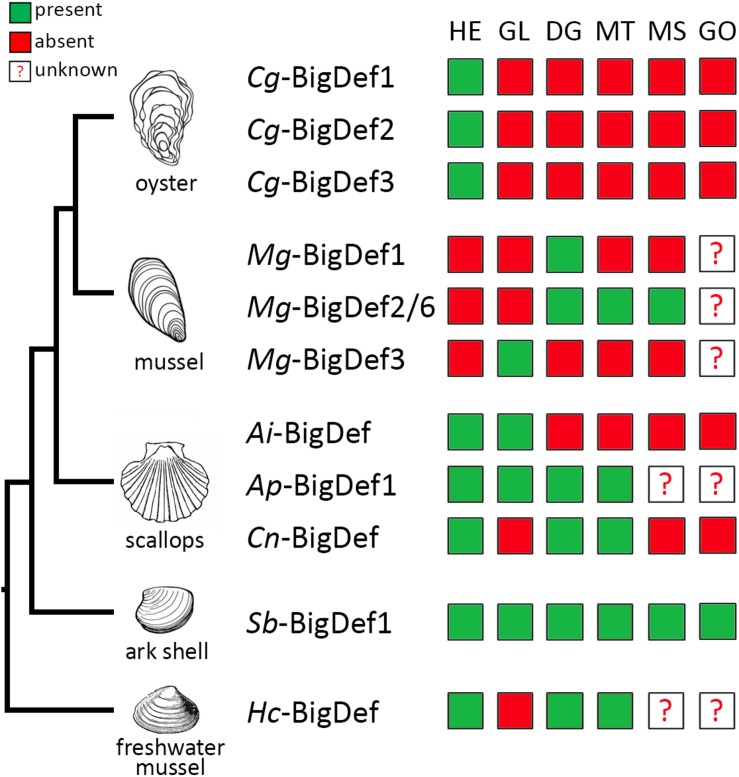
Expression profiles of big defensin genes among different tissues from bivalve mollusks. Big defensin expression in hemocytes (HE), gills (GI), digestive gland (DG), mantle (MT), muscle (MS), and gonad (GO) tissues from the oyster *Crassostrea gigas*; the mussel *Mytilus galloprovincialis*; the scallops *Argopecten irradians, A. purpuratus*, and *Chlamys nobilis*; the ark shell *Scapharca broughtonii* and the freshwater mussel *Hyriopsis cumingii*, is represented in a color code schema. Green squares indicate detection of big defensin transcripts; red squares represent that the expression is undetected. Question mark indicates that the expression in those tissues has not been analyzed.

Big defensins display highly different patterns of expression in terms of tissues and inherent array of genes in one species. According to species, big defensins are expressed in hemocytes or epithelia, tissues which play important roles in immunity. The expression of big defensins is specific to hemocytes in oysters (15) and horseshoe crabs (12). In contrast, in mussels (16, 35), scallops (30, 31) and clams (34), big defensins are mainly expressed in epithelial tissues (Figure 6); their expression in hemocytes is either undetectable or lower than in other tissues. Tissue-specific expression of big defensin genes is sometimes observed. For instance, in the Mediterranean mussel *M. galloprovincialis, Mg*-BigDef1, *Mg*-BigDef3, and *Mg-*BigDef6 are constitutively expressed in the digestive gland, gills and mantle, respectively (16). Therefore, it can be speculated that mussel big defensin genes carry distinct biological functions and control the host-microbiota homeostasis at the main epithelial surfaces. While the reasons behind the marked differences in tissue specificity observed among different species is still unknown, one possible explanation may be sought in the functional replacement with other AMPs, such as mytilins, myticins and invertebrate-type defensins in mussel hemocytes.

In addition, big defensin genes respond differently to environmental stimuli. For instance, in healthy *C. gigas* oysters, *Cg*-BigDef1 and *Cg*-BigDef2 are expressed at very low basal levels whereas *Cg*-BigDef3 is constitutively expressed, and only *Cg*-BigDef1 and *Cg*-BigDef2 are induced in response to bacterial challenge (15) (Figure 6), mirroring the case of other human β-defensins (53). While *Cg*-BigDef3 does not respond to bacterial challenge (15) its expression is repressed by OsHV-1 (Ostreid herpesvirus type 1) viral infection (54). These different regulatory patterns strongly suggest different roles in oyster immunity. In the ark shell *S. broughtonii* and the freshwater mussel *Hyriopsis cumingii*, *Sb*-BigDef1 and *Hc-*BigDef are expressed constitutively in different tissues and overexpressed in some of them after challenge (34, 35). In scallops, a single big defensin gene is expressed at low levels in hemocytes compared with other tissues such as mantle or gills, but it is overexpressed in hemocytes and epithelia after *Vibrio* challenge (30). A feature common to all big defensins is the lack of response to damage-associated molecular patterns, triggered by wounding or injection of sterile seawater.

Further supporting an important role in immunity, recent studies have shown that big defensins are regulated by the NF-κB/Rel pathway, as revealed by transcriptional knockdown of genes implicated in the pathway. Specifically, the silencing of *Cg*Rel expression in the oyster *C. gigas* (55) and the inhibitor of NF-kB transcription factor in the scallop *Argopecten purpuratus* (56) indicates the participation of NF-κB/Rel pathway in the regulation of big defensin expression.

In mussels and oysters, a third and important degree of variability in big defensin expression is introduced by PAV (see section above), with an extreme inter-individual variability in the expression of big defensin isoforms encoded by different genes and/or alleles (51).

Whether and how tissue-expression, gene-regulation and PAV affecting big defensins may impact the biology and ecology of mollusks remains to be determined. To better understand the functions of big defensins more research is now needed at a protein level. Until now this has been hampered by limitations in producing big defensins and, as a consequence, specific antibodies. Recently, in the scallop *A. purpuratus*, *Ap-*BigDef1 was localized not only inside hemocytes but also in the digestive gland, mantle and gill tissues from challenged scallops (30). With the recent developments to produce big defensins in large amounts (21), new perspectives are now open for understanding the biology of these HDPs, from tissue distribution to function across multiple species.

## Functional Consequences of Big Defensin Molecular Evolution

An amazing feature of big defensin molecular evolution discussed in this review is their loss, pseudogenization and molecular evolution toward novel forms, including β-defensins, in diverse phyla (Ecdysozoa, Deuterostomia) (Figure 3), as opposed to their conservation and major diversification in Lophotrochozoa, particularly in mollusks.

### Potential Trade-Offs Between Immune Function and Host Fitness

In species harboring the canonical big defensin structure (horseshoe crab, amphioxus, scallop and oyster), big defensins play immune functions: actually, they have antimicrobial activities at the physiological salt concentrations of their marine host, indicating they likely contribute to the defense against infections (12, 14, 17, 21). The oyster *Cg-*BigDef1 remains the only big defensin produced in sufficient amounts to establish a large activity spectrum (21). It shows a broad range of bactericidal activities against reference, environmental, and clinical strains, including strains multiresistant to antibiotics. Supporting further a role in controlling infections, *Cg-*BigDef1 is one of the few AMPs of *C. gigas* having significant antimicrobial activities against *Vibrio* species pathogenic for oysters (21, 57).

The loss of big defensins in several classes of Ecdysozoa and Deuterostomia suggests that the maintenance of these HDPs could be highly costly for their hosts. AMP gene loss and pseudogenization can result from a high fitness cost either because AMPs can damage host tissues or kill beneficial components of the host microbiota (58). As a consequence, for species exposed to low infection pressures, AMPs can be readily lost or accumulate mutations that compromise their function (58). This trade-off hypothesis is supported by the presence of a high number of pseudogenes in bivalves. In species where only β-defensin-like peptides but no big defensins are found, a likely hypothesis is an evolution toward other functions. In deuterostomes, this is exemplified by human β-defensins, which lack direct antimicrobial activity at physiological salt concentrations but act as key immunomodulators controlling infections (59). The ability of AMPs to carry multiple functions beyond antimicrobial is illustrated by myticins, another family of cysteine-rich peptides found in mussels (60, 61), or macins, CSαβ-containing peptides found in a number of invertebrates (62). The coexistence of both canonical big defensins and non-canonical peptides lacking the N-terminal region in some bivalve mollusks (*R. philippinarum* and *D. rostriformis*) may be indicative of an ongoing process of neofunctionalization.

We have earlier hypothesized that strong selection pressures imposed by marine environments may explain the scattered distribution of big defensins across animal species, mostly in marine species (21). Indeed, while most β-defensins are salt-sensitive (63), big defensins retain antimicrobial activity at high salt concentration and this property was assigned to the hydrophobic N-terminal domain lost during evolution toward big defensins (21). However, this view is partly questionned by our identification of canonical big defensins in different freshwater bivalve and gastropod species and by the observation that big defensins are absent in many large phyla of marine organisms (e.g., echinoderms and tunicates). As discussed in the previous sections, the current discontinuous distribution of big defensins may appear consistent with the massive genome reduction events that have led to the loss of several thousand genes in multiple metazoan lineages (64, 65). At the same time, it is certainly noteworthy that, to date, no big defensin has ever been identified in terrestrial species. Therefore, while marine habitats cannot be considered as the only drivers of the retention of big defensins, it is likely that the evolutionary scenario we have highlighted in this review is the product of a combination between multiple ecological and evolutionary factors whose relative weight could be only addressed in the future though advanced phylogenomic profiling studies.

### Conserved and Diversified Molecular Patterns in Canonical Big Defensins

**FIGURE 7:**
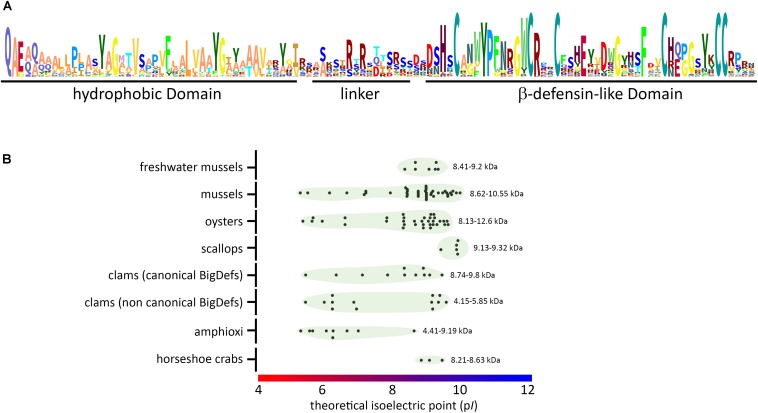
Conserved molecular patterns and diverse biophysical properties of big defensins. **(A)** Schematic view (sequence logo) of the conserved amino acids found in big defensins. **(B)** Variability of isoelectric point (p*I*) and molecular weight (kDa) among big defensins from different taxa. Black dots represent single big defensin sequences from each taxon, and green circles indicate the dispersion of the p*I* values (indicated on the *X* axis). At the right side of each group, the range of deduced molecular weight is presented (kDa).

**FIGURE 8:**
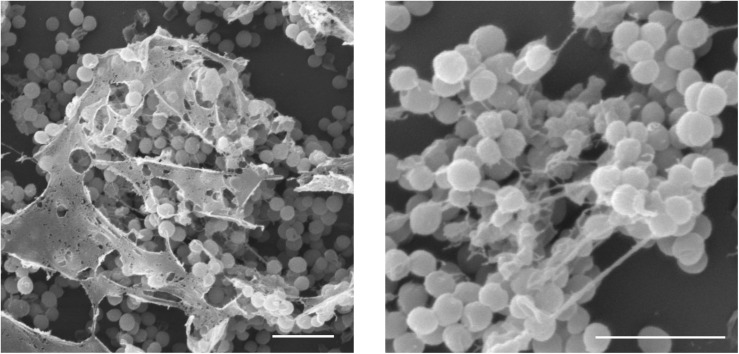
Bacterially triggered assembly of *Cg*-BigDef1 into nanonets. Large and branched fibers entrapping *Staphylococcus aureus* are observed by scanning electron microscopy when *S. aureus* SG4511 is exposed to *Cg*-BigDef1. The same observations are made with *S. aureus* exposed to the N-terminal domain alone (21). Bars represent 3 μm.

The ancestral N-terminal domain preserved in canonical big defensins does not present any homology with known sequences outside this AMP family, questioning its role in big defensin mechanism of action. Remarkably, it has a well conserved sequence that retains hydrophobic properties (Figure 7), suggesting a similar function across big defensins. It has been suggested that the insertion of the N-terminal domain into membranes is involved in the antimicrobial activity of the horseshoe crab *Tt*-BigDef (20). However, such a membrane activity was shown to be uncoupled to the activity of oyster *Cg-*BigDef1. Instead, *Cg-*BigDef1 N-terminal domain drives bacteria-triggered peptide assembly into nanonets that entrap and kill *Staphylococcus aureus* (Figure 8). The hydrophobicity of this domain would be essential to nanonet formation and salt-stable antimicrobial activity (21). Such nanonets were earlier identified for human α-defensin 6 (HD6) (66) and human β-defensin 1 (hDB1) (67). They were recently observed for the scallop *Ap-*BigDef1 (68). This indicates that this property is shared by different *trans-*defensins and among them distinct big defensins regardless of sequence diversity. It suggests a key role in their mechanism of action.

In canonical big defensins, sequence diversification is observed at different levels: (i) amino acid composition and (ii) length of the flexible linker that connects the two domains. Changes in amino acid composition strongly affect the charge of the big defensins, which can vary from anionic to cationic (Figure 7 and Supplementary Table S1), with potential consequences on interactions with microorganisms. However, up to now, there is no evidence that electrostatic interactions are involved in the mechanism of action of big defensins. More unexpectedly, surface properties of big defensins were shown to drastically vary with the length of the flexible linker that connects the two domains. Indeed, the linker length induces a different orientation of the N- and C-terminal domains (Figure 2). As a consequence, *Tt-*BigDef is amphipathic whereas *Cg-*BigDef1 is hydrophobic (21). This likely has major consequences on the interaction of big defensins with prokaryotic and eukaryotic cells. Linker length varies also significantly between big defensins at an intraspecific level (Supplementary Figure S2). The role of linkers in big defensin 3D structures and activities remains an important aspect to be explored.

It was hypothesized that antimicrobial activity of big defensins could be carried by their β-defensin-like domains, while the N-terminal domains would promote close contact with bacteria (21). This raises another important unsolved question. Are the two domains cleaved apart upon interaction with bacteria? Although big defensin cleavage has only been evidenced *in vitro* (12), it is likely that the basic and di-basic sites often present on big defensin flexible linkers (Figure 4) are accessible to bacterial proteases, which can trigger the release of the β-defensin like-domains at the close vicinity of the microbes. If so, the N-terminal domains of big defensins could serve as a cargo to release active and concentrated peptides on microorganisms, the specificity of which could be carried by the β-defensin-like domains and vary with the sequence diversity of these domains.

## Big Defensins at the Host-Microbiota Interface

As a compromise between effective defense responses and optimal host fitness, the AMP/HDP repertoire of a given animal species must inhibit or kill pathogenic microbes without seriously unbalancing the host-associated microbiota nor damaging host tissues (58). The evolutionary diversification of AMP/HDP genes and families disclosed so far in bivalve mollusks and available functional data indicate unexpected individual differences (44), multiple action modes (20, 53) and even the possible maintenance of isoforms or alleles coding poorly effective antimicrobials (69). In essence, healthy bivalves can be regarded as a dynamic assemblage of host-microbe interactions in which the host maintains tolerable amounts of symbionts or commensals and even opportunists or parasites (70). Environmental factors such as temperature and salinity changes, microorganism blooms and inappropriate farming practices can break such homeostasis, leading to diseases and death (71, 72).

The unique gene landscapes and expression profiles of big defensins and other AMPs support a tight control of microbiota in healthy oysters and mussels (3, 48). However, the impact of big defensins on the host microbiota has been poorly studied until now. Recently, the Ostreid herpesvirus type 1 (OsHV-1) was shown to suppress the expression of AMPs, in particular big defensins (both the inducible *Cg*-BigDef1/2 and the constitutively expressed *Cg-*BigDef3). This led to a fatal dysbiosis characterizing the Pacific oyster mortality syndrome (POMS) (54, 73). This is certainly the best indication that big defensins could be key players in the interaction with the host microbiota. In agreement, important microbiota changes were observed upon induction of big defensin in scallops (74), which further suggests a role for big defensins in host-microbiota homeostasis.

This view of big defensin peptides as key defense effectors in mollusks is consistent with data reported for other invertebrate AMPs that select species-specific microbiota (75), control the hemolymph microbiota (76) or control pathogenic infections (77).

## Translational Insights From Big Defensins

### Big Defensins in Marker-Assisted Selection of Bivalve Broodstock

Either in the whole or as an archetypal gene family, species-specific AMP repertoires were shown to determine host aptitude for pathogen-resistance. This was recently illustrated in *Drosophila melanogaster* through experimental knockout of ten known AMP genes (78). Therefore, AMPs could serve as a proxy for the immune system competence in a marker-assisted selection of bivalve broodstock.

Individual phenotypes are shaped by complex gene-environment interactions and the host immune response is a metric (not dichotomous) phenotypic trait resulting from the action of several genes, each one subjected to multiple regulation levels, with specific alleles generating additive or non-additive genetic effects, such as dominance or epistasis. Following the hypothesis of polygenic and mostly additive genetic effects (79), a low heritability of the “response to infection” provided by a single defense molecule would not be surprising. Breeding programs in *C. gigas* produced oyster families with different (stable) levels of resistance to POMS (80, 81), triggered by the OsHV-1 μvar virus. Resistance was identified as heritable (80, 82) with some candidate markers having a role in distinct antiviral pathways (79, 83). Big defensins and other AMPs were not identified as associated with resistance to POMS, probably because the bacteremia comes as a secondary infection in this virus-induced immunosuppressive disease. Due to their potent activities against vibrios, it can be speculated that AMPs will rather arise as good resistance markers in disease where bacteria (e.g., *Vibrio aestuarianus*) are the primary infectious agents (84).

The mounting demand of support to fish and shellfish aquaculture requires knowledge-based solutions and oriented research work. Sequence diversity, salt-stable antimicrobial activity and gene presence/absence variation indicate bivalve big defensins as key candidates to be considered among other AMPs/HDPs, in the assessment of host immune-competence for the genetic improvement of farmed stocks.

### Big Defensin-Inspired Nanonets

Antimicrobial resistance is a major concern for public health worldwide (85). Antibiotics have been extensively used for decades, generating strong selective pressures on microorganisms. This has selected resistant genotypes that currently threaten the sustainability of the “modern medicine” (86, 87). Even though AMPs are often seen as possible alternatives to antibiotics and huge research efforts are made to isolate new AMPs (88), resistance and tolerance to AMPs, i.e., their capacity to survive a transient exposure to AMPs (89) are already well described phenomena (90–93). This highlights the need to develop new anti-infectives less prone to induce resistance (94).

The diversity of AMP structures and the multiple functions they harbor as HDPs offer a platform to design new drugs (95). A few examples in the literature have shown that HDPs from eukaryotic organisms show considerable advantage over antibiotics as they induce only limited resistance (low increase in mutation rate and horizontal gene transfer) (96). Over the past years, the immunomodulatory functions of AMPs/HDPs have been successfully exploited to develop effective anti-infectives with very limited risk to induce resistance (97).

With the recent discovery of nanonet formation as an indirect way by which AMPs/HDPs control infections by entrapping bacteria without necessarily killing them (21, 66), a new field is open to design new drugs. The present review illustrates that the hydrophobic N-terminal domain of big defensins, which carries the capacity to form nanonets upon contact with bacteria, has hydrophobic and nanonet-forming properties. Engineering of nanonet-forming AMPs, in which the N-terminal domain of big defensins is used as a cargo to deliver AMPs with different activities in close contact with microorganisms, is promising in many regards. Such multi-domain antimicrobials should provide an important advantage in terms of AMP resistance as (i) their activity depends on the nanonet formation rather the only interaction with a receptor, which can be easily mutated, and (ii) combination of peptides (cocktails) with multiple bacterial targets have already proved to limit resistance (98). It is also important as salt-stable AMPs are currently needed to treat infections associated to cystic fibrosis, a disease in which salt-treatment is used to control infections. It can be argued that nanonets (peptide aggregates) present a risk of having toxic effects, as known for amyloid fibers involved in ageing (Alzheimer’s disease) (99). The current literature shows that nanonets are an evolutionary-conserved defense strategy in AMP families from highly divergent phyla (21, 66). It is likely that, if toxic, this defense mechanism would have been counter-selected. Moreover, no toxicity was observed for *Cg*-BigDef1 on human cell lines (21). For all these reasons, we believe that novel translational insights can be gained through the design of novel antimicrobials inspired by big defensins.

## Conclusion

Big defensins have a complex and fascinating evolutionary history in the animal kingdom, with gene losses in many species of Ecdysozoa and Deuterostomia as opposed to major diversification in Lophotrochozoa. With two species where the canonical big defensins co-exist with shorter β-defensin-like peptides (non-canonical big defensins), this review highlights an ongoing evolutionary process and supports the hypothesis that β-defensins derived from big defensins. The massive expansion and diversification of β-defensins in Deuterostomia echoes to big defensin molecular evolution in Lophotrochozoa, which appears to be the product of independent lineage-specific gene tandem duplications followed by a rapid molecular diversification, with an additional layer of complexity provided by the PAV phenomenon. Like β-defensins, big defensins have therefore likely acquired novel functions, which remain to be uncovered. Key features conserved in canonical big defensins highlight the importance of the hydrophobic N-terminal domain, which drives the formation of nanonets and plays an important role in maintaining big defensin antimicrobial activity at high salt concentrations, an additional reason to consider them as animal health markers. Having maintained broad and salt-stable antimicrobial activities, and being active against multi drug resistant bacteria, the ancestral (canonical) structure of big defensins also inspires the nature-based design of novel therapeutics.

## Author Contributions

All authors have contributed to the writing of the review article, they agreed to be accountable for all aspects of the work in ensuring that questions related to the accuracy or integrity of any part of the work are appropriately investigated and resolved, and they provided approval for publication of the content.

## Conflict of Interest

The authors declare that the research was conducted in the absence of any commercial or financial relationships that could be construed as a potential conflict of interest.

## Footnotes

1 https://blast.ncbi.nlm.nih.gov/Blast.cgi
