# Supplementary material for: Functional Insights From the Evolutionary Diversification of Big Defensins
Source: Front Immunol. 2020 Apr 30;11:758. doi: 10.3389/fimmu.2020.00758 (PMC7203481; doi:10.3389/fimmu.2020.00758)
Supplement: Supplementary file 2 [file Data_Sheet_1.docx]

**
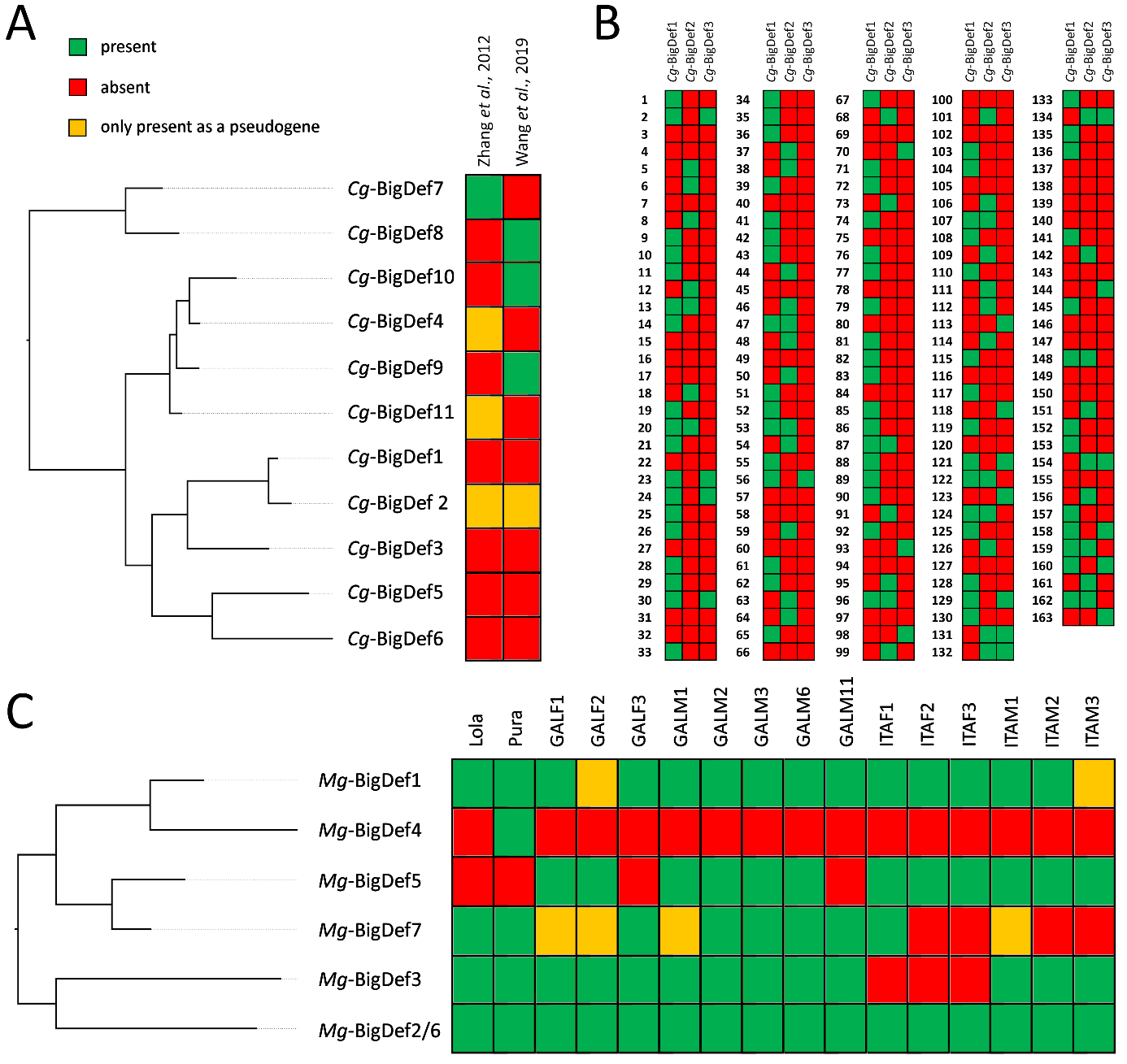
**

**Supplementary Figure S1**. Patterns of gene presence/absence variability (PAV) across individuals. (**A**) Simplified phylogeny and PAV of big defensin genes in *Crassostrea gigas*, based on data gathered from genome assemblies (Zhang *et al*., 2012; Wang *et al*., 2019). (**B**) Complete PAV profiles of *Cg*-BigDef1, *Cg*-BigDef2 and *Cg*-BigDef3 in 163 *C. gigas* individuals. Data extracted from Rosa *et al*. (2015). (**C**) Simplified phylogeny and PAV of big defensin genes in *Mytilus galloprovincialis*, based on the analysis of the genome of 16 different individuals from Gerdol *et al*. (2019). “Lola” indicates the reference genome (a female mussel from Galicia), “Pura” indicates the individual sequenced in a previous effort (Murgarella *et al*., 2014) whereas the “GAL” and “ITA” prefixes indicate mussels collected in Galicia and Italy, respectively. “M” and “F” indicate male and female mussels, respectively.

References:

Gerdol M, Moreira R, Cruz F, Gómez-Garrido J, Vlasova A, Rosani U, Venier P, Naranjo-Ortiz MA, Murgarella M, Balseiro P, et al. Massive gene presence/absence variation in the mussel genome as an adaptive strategy: first evidence of a pan-genome in Metazoa. bioRxiv (2019)781377. doi:10.1101/781377

Murgarella M, Corvelo A, Alioto T, Novoa B, Figueras A, Posada D, Canchaya C. Genomic characterization of the aquaculture resource Mytilus galloprovincialis. Front Mar Sci (2014) 1: doi:10.3389/conf.FMARS.2014.02.00113

Rosa RD, Alonso P, Santini A, Vergnes A, Bachère E. High polymorphism in big defensin gene expression reveals presence-absence gene variability (PAV) in the oyster *Crassostrea gigas*. *Dev Comp Immunol* (2015) **49**:231–238. doi:10.1016/j.dci.2014.12.002

Wang X, Xu W, Wei L, Zhu C, He C, Song H, Cai Z, Yu W, Jiang Q, Li L, et al. Nanopore Sequencing and De Novo Assembly of a Black-Shelled Pacific Oyster (Crassostrea gigas) Genome. Front Genet (2019) 10:1211. doi:10.3389/fgene.2019.01211

Zhang G, Fang X, Guo X, Li L, Luo R, Xu F, Yang P, Zhang L, Wang X, Qi H, et al. The oyster genome reveals stress adaptation and complexity of shell formation. Nature (2012) 490:49–54. doi:10.1038/nature11413
