# Supplementary material for: Functional Insights From the Evolutionary Diversification of Big Defensins
Source: Front Immunol. 2020 Apr 30;11:758. doi: 10.3389/fimmu.2020.00758 (PMC7203481; doi:10.3389/fimmu.2020.00758)
Supplement: Supplementary file 3 [file Data_Sheet_2.docx]

**
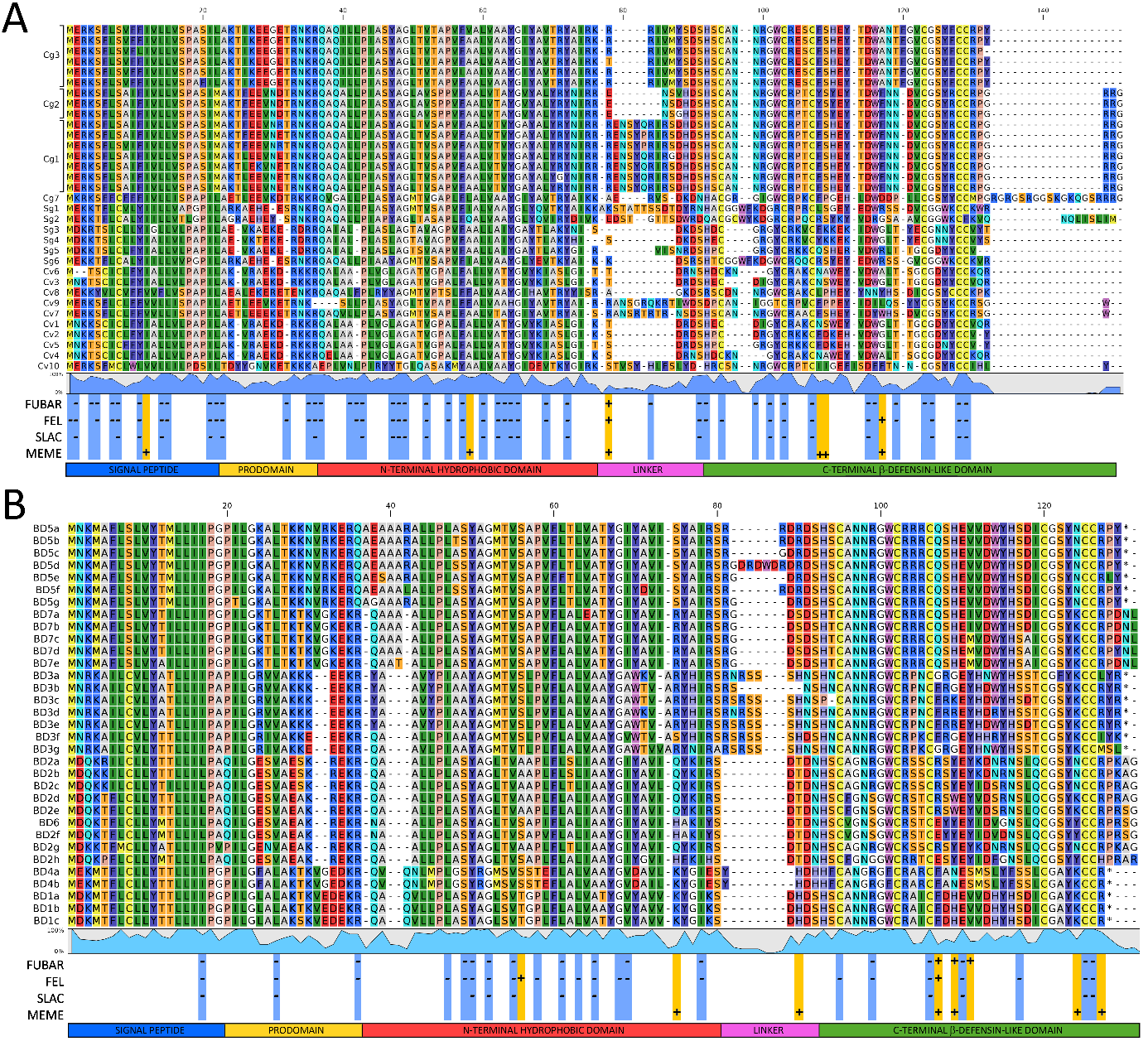
**

**Supplementary Figure S2**. Multiple sequence alignment of big defensins identified in the genomes from the oysters *Crassostrea gigas* (*Cg*1 to -3 and -7), *Crassostrea virginica* (*Cv*1 to -10) and *Saccostrea glomerata* (*Sg*1 to -6) (**A**) and from the Mediterranean mussel *Mytilus galloprovincialis* (BD1 to -7) (**B**). The different allelic variants identified in *C. gigas* by (Rosa *et al*., 2015) are also shown (variants encoding identical precursor proteins have been removed). Residues under negative and positive selection, detected with the FUBAR, FEL, SLAC and MEME tools included un the DataMonkey Adaptive Evolution Server (Weaver *et al*., 2018) are highlighted in blue and orange respectively. Note the various lengths of the linkers connecting the two big defensin domains.

References:

Rosa RD, Alonso P, Santini A, Vergnes A, Bachère E. High polymorphism in big defensin gene expression reveals presence-absence gene variability (PAV) in the oyster *Crassostrea gigas*. *Dev Comp Immunol* (2015) **49**:231–238. doi:10.1016/j.dci.2014.12.002

Weaver S, Shank SD, Spielman SJ, Li M, Muse SV, Pond SLK. Datamonkey 2.0: A Modern Web Application for Characterizing Selective and Other Evolutionary Processes. *Mol Biol Evol* (2018) **35**:773–777. doi.org/10.1093/molbev/msx335
